# Supplementary material for: MetaRibo-Seq measures translation in microbiomes
Source: Nat Commun. 2020 Jun 29;11:3268. doi: 10.1038/s41467-020-17081-z (PMC7324362; doi:10.1038/s41467-020-17081-z)
Supplement: Supplementary file 10 — Supplementary Data 7 [file 41467_2020_17081_MOESM10_ESM.zip › File2/Confidence_High_Taxonomy/107629_out.krona.html]

Javascript must be enabled to view this page.

members
magnitude
magnitudeUnassigned
count
unassigned
taxon
rank

107629\_out

119

2
superkingdom
119

976
119
phylum

class
119
200643

119
order
171549

119
family
815

2

SRS143085\_contig\_number\_contig-100\_583.584SRS148970\_contig\_number\_contig-100\_1524.1525
119
genus
816

817

SRS011084\_contig\_number\_27744SRS011239\_contig\_number\_7545SRS012902\_contig\_number\_703SRS013098\_contig\_number\_6559SRS013158\_contig\_number\_583SRS013215\_contig\_number\_119SRS013476\_contig\_number\_23241SRS014459\_contig\_number\_7067SRS014613\_contig\_number\_21424SRS014736\_contig\_number\_7038SRS015065\_contig\_number\_10873SRS015190\_contig\_number\_1194SRS015264\_contig\_number\_17297SRS015578\_contig\_number\_422SRS015854\_contig\_number\_174SRS015890\_contig\_number\_468SRS015960\_contig\_number\_12105SRS016095\_contig\_number\_1155SRS016132\_contig\_number\_33SRS016267\_contig\_number\_275SRS016495\_contig\_number\_9699SRS017247\_contig\_number\_7979SRS017433\_contig\_number\_458SRS017521\_contig\_number\_15319SRS017622\_contig\_number\_140SRS018313\_contig\_number\_11292SRS018541\_contig\_number\_889SRS018623\_contig\_number\_8379SRS018656\_contig\_number\_12074SRS018817\_contig\_number\_316SRS018888\_contig\_number\_788SRS018984\_contig\_number\_1241SRS019267\_contig\_number\_5255SRS019286\_contig\_number\_contig-100\_3780.3781SRS019397\_contig\_number\_146SRS019601\_contig\_number\_3937SRS019787\_contig\_number\_1084SRS020233\_contig\_number\_424SRS020869\_contig\_number\_8354SRS021484\_contig\_number\_3683SRS022524\_contig\_number\_9323SRS023346\_contig\_number\_1254SRS023583\_contig\_number\_11803SRS023829\_contig\_number\_54SRS023971\_contig\_number\_221SRS024075\_contig\_number\_538SRS024132\_contig\_number\_2708SRS024388\_contig\_number\_606SRS024549\_contig\_number\_1244SRS043411\_contig\_number\_3857SRS043701\_contig\_number\_535SRS046502\_contig\_number\_contig-100\_819.77799SRS046712\_contig\_number\_323SRS048981\_contig\_number\_128SRS049402\_contig\_number\_1048SRS050299\_contig\_number\_379SRS051610\_contig\_number\_1516SRS053214\_contig\_number\_11787SRS053573\_contig\_number\_726SRS055966\_contig\_number\_478SRS056259\_contig\_number\_7974SRS057717\_contig\_number\_696SRS058723\_contig\_number\_793SRS063040\_contig\_number\_46356SRS063127\_contig\_number\_954SRS063190\_contig\_number\_1058SRS063985\_contig\_number\_contig-100\_498.151153SRS064276\_contig\_number\_436SRS074964\_contig\_number\_800SRS075021\_contig\_number\_67SRS075398\_contig\_number\_331SRS075773\_contig\_number\_9964SRS075878\_contig\_number\_325SRS076804\_contig\_number\_1665SRS077194\_contig\_number\_1504SRS077335\_contig\_number\_648SRS077589\_contig\_number\_666SRS077730\_contig\_number\_153SRS097920\_contig\_number\_7560SRS098571\_contig\_number\_52374SRS098644\_contig\_number\_9088SRS098655\_contig\_number\_1017SRS098717\_contig\_number\_161SRS098881\_contig\_number\_13514SRS104036\_contig\_number\_3658SRS1041039\_contig\_number\_3549SRS104311\_contig\_number\_11568SRS104485\_contig\_number\_564SRS104636\_contig\_number\_539SRS1054928\_contig\_number\_958SRS1055043\_contig\_number\_9033SRS1055069\_contig\_number\_523SRS1055099\_contig\_number\_187SRS140492\_contig\_number\_1840SRS140513\_contig\_number\_8432SRS140645\_contig\_number\_contig-100\_501.501SRS142599\_contig\_number\_1653SRS143342\_contig\_number\_9445SRS143417\_contig\_number\_14938SRS143598\_contig\_number\_81SRS144135\_contig\_number\_30593SRS144362\_contig\_number\_691SRS144506\_contig\_number\_20899SRS146812\_contig\_number\_57236SRS147022\_contig\_number\_1114SRS147139\_contig\_number\_11078SRS147272\_contig\_number\_278SRS147377\_contig\_number\_3336SRS147557\_contig\_number\_2355SRS147653\_contig\_number\_2044SRS148091\_contig\_number\_9766SRS148159\_contig\_number\_18168SRS149075\_contig\_number\_5903SRS893279\_contig\_number\_574SRS893288\_contig\_number\_2048SRS893342\_contig\_number\_218SRS971275\_contig\_number\_14098
117
species
